# The Association Between Cyberbullying Victimization and Suicidal Ideation Among Chinese College Students: The Parallel Mediating Roles of Core Self-Evaluation and Depression

**DOI:** 10.3389/fpsyt.2022.929679

**Published:** 2022-06-30

**Authors:** Xiaowei Chu, Sumin Yang, Zhaoxing Sun, Min Jiang, Ruibo Xie

**Affiliations:** ^1^College of Teacher Education, Zhejiang Normal University, Jinhua, China; ^2^Key Laboratory of Intelligent Education Technology and Application of Zhejiang Province, Jinhua, China

**Keywords:** cyberbullying victimization, suicidal ideation, core self-evaluation, depression, college students

## Abstract

With the rapid development of science and technology, the Internet has formed a new form of aggression, which is called cyberbullying. Many studies have demonstrated that cyberbullying can cause serious damage to the physical and mental health of Chinese college students, such as depression and suicide. The main purpose of this study was to investigate the relationship between cyberbullying victimization and suicidal ideation and the parallel mediating roles of core self-evaluation and depression. A questionnaire was used to measure the research variables in this study among 1,509 college students. The results indicated that: After controlling for participants' gender, age, family structure, and family economic status, cyberbullying victimization significantly and positively related to suicidal ideation. Core self-evaluation and depression separately mediated the relationship between cyberbullying victimization and suicidal ideation. The mediating effect of depression was stronger than that of core self-evaluation. The findings support a parallel mediation model of the relationship between cyberbullying victimization and suicidal ideation. Our study may help to develop interventions and prevention measures for college students who experienced cyberbullying victimization.

## Introduction

With the development of technology, the Internet has become common in homes, schools, and workplaces. According to the latest “49th China Internet Network Development Statistics Report” in February 2022 released by China Internet Network Information Center, the number of Chinese Internet users is 1.032 billion (1). Although the Internet provides us with many conveniences, it also produces many bad consequences, such as cyberbullying. It is a new type of bullying, which is defined as “any behavior performed through electronic or digital media by individuals or groups that repeatedly communicates hostile or aggressive messages intended to inflict harm or discomfort on others” [(2), p. 278]. In China, given that university students are the most active users of electronic technology, it is important and essential to study cyberbullying among Chinese university students. A review reported that the victimization rate and the perpetration rate of cyberbullying ranged from 14.6 to 52.2% and 6.3 to 32.0%, respectively. In China, the prevalence of cyberbullying victimization ranged from 6.0 to 46.3% (3).

Suicide is one of the most common causes of death among adolescents or young adults all over the world (4, 5). However, the incidence of suicidal attempts is higher than the incidence of accomplished suicide, and the occurrences of suicidal ideation are even more prevalent (6). What is worse is that suicidal ideation could cause many negative consequences, such as elevated psychological problems, succeeding suicide attempts, and elevated mortality (7–10). Therefore, attention to risk factors (e.g., cyberbullying victimization) for suicidal ideation is necessary. This would provide preventive measures for individuals' suicidal behaviors.

One study found that cyberbullying could cause a significant increase in suicidal ideation, suicidal attempts, and suicidal mortality (11). Empirical studies have demonstrated a positive relationship between cyberbullying victimization and suicidal ideation in adolescents and college students (12–14). In China, Luo et al. (15) demonstrated that being cyberbullied was significantly associated with suicidal ideation among college students. However, the explanatory mechanism for the relation between cyberbullying victimization and suicidal ideation remains unclear. Therefore, to bridge this gap, the main purpose of this study was to explore the indirect effects (i.e., how cyberbullying victimization is associated with suicidal ideation) in the relationship between cyberbullying victimization and suicidal ideation among university students. Specifically, this study investigated core self-evaluation and depression as two possible parallel mediators and compared the mediating effects of two parallel mediators. This is helpful to understand the *ways* how cyberbullying victimization predicts suicidal ideation, and provide a theoretical basis for cyberbullying interventions on the side of victims.

### Mediating Role of Core Self-Evaluation

Why is cyberbullying victimization positively associated with suicidal ideation? One possible explanation is core self-evaluation. Judge et al. (16) define core self-evaluations as fundamental assessments that individuals hold about themselves, reflecting people's perceptions of themselves. Researchers also point out that core self-evaluation consists of four traits: self-esteem, generalized self-efficacy, locus of control, and emotional stability (17). According to the Psychological Mediation Framework (PMF), negative events or situations affect individuals' psychology or behavior by influencing their internal states such as attitudes and cognition (18). The model consists of two processes. One is the distal stress process, which refers to exposure to negative life events or situations, including bullying and rejection (19). The other is a proximal stress process, which refers to individuals' negative attitudes and cognition when exposed to negative life events or situations. In the cognitive processing of externally negative information, individuals subjectively believe that others will reject, discriminate and treat them unfairly (20). In this sense, they may underestimate their self-values (e.g., low self-esteem, lack of self-confidence, and unstable emotions), which may further increase the likelihood of experiencing suicidal ideation.

Cyberbullying victimization may hurt an individual's core self-evaluation. Individuals' core self-evaluations are characterized by both external dependence and contextuality (21). The negative life events or situations (e.g., cyberbullying) provide important external information to non-adaptive attitudes or perceptions (e.g., low self-evaluation). The experience of being bullied may further increase the victims' negative evaluation and reduce their positive evaluation toward themselves. Research also suggested that cyberbullying victimization can reduce the victim's positive self-evaluation (22). Besides, low core self-evaluation may be a risk factor for suicidal ideation. Individuals with low core self-evaluation are more likely to interpret information negatively when processing it, further affecting their own psychological and behavioral adaptation (23). Numerous studies have found that low core self-evaluation was a risk factor predicting suicidal ideation (22, 24). For instance, in Jones et al.'s (25) study, low self-esteem has been shown to significantly predict suicidal ideation. Denneson et al. (26) found that low self-efficacy increased the risk of suicidal ideation. Neacsiu et al. (27) also demonstrated that emotional regulation difficulties predicted suicidal ideation.

No empirical evidence directly supports the mediating role of core self-evaluation between cyberbullying victimization and suicidal ideation. However, some previous research can provide indirect evidence. Research has revealed core self-evaluation as a mediator in the relationship between stressful life events (e.g., parental conflict, weight bias, peer alienation, and childhood psychological maltreatment) and negative outcomes (28–31). For example, core self-evaluation has been found to mediate the relationship between parental conflict and Internet addiction (28). A study demonstrated core self-evaluation as a mediator in the relationship between peer alienation and cyber deviant behaviors in adolescents (30). Besides, Wang et al. (31) found a mediating effect of core self-evaluation between childhood psychological maltreatment and depression. Thus, the present study hypothesized that core self-evaluation would mediate the relationship between cyberbullying victimization and suicidal ideation.

### Mediating Role of Depression

Depression is another possible mechanism explaining the relationship between cyberbullying victimization and suicidal ideation. Numerous cross-sectional studies have revealed that individuals who have been cyberbullied may suffer from depressive symptoms (32–35). Longitudinal studies also found that the experience of cyberbullying victimization was longitudinally associated with depression (36–38). Meta-analyses indicated that cyberbullying victimization was positively associated with depression (13, 39). Besides, depression is an obvious risk factor for suicidal ideation. Some studies have shown that mental disorders, especially major depression, are risk factors for suicidal ideation (24, 40–42). According to the interpersonal theory of suicide, perceived burdensomeness and failed belongingness are two risk factors for suicide (43). Perceived burdensomeness refers to the mistaken belief that he/she is a burden in the lives of others, including self-hatred. Failed belongingness refers to the absence of good interpersonal relationships. Some researchers have shown that depression is positively correlated with perceived burdensomeness, meaning that people who are depressed have higher levels of perceived burdensomeness. This may increase their suicidal thoughts. Furthermore, previous studies have demonstrated the mediating effect of depression between bullying victimization and suicide (14, 44). For instance, Mitchell et al. (14) demonstrated that depression mediated the relation between the intensity of being cyberbullied and suicide ideation in college students. Therefore, this study predicted that depression would mediate the role of cyberbullying victimization and suicidal ideation.

**Figure 1:**
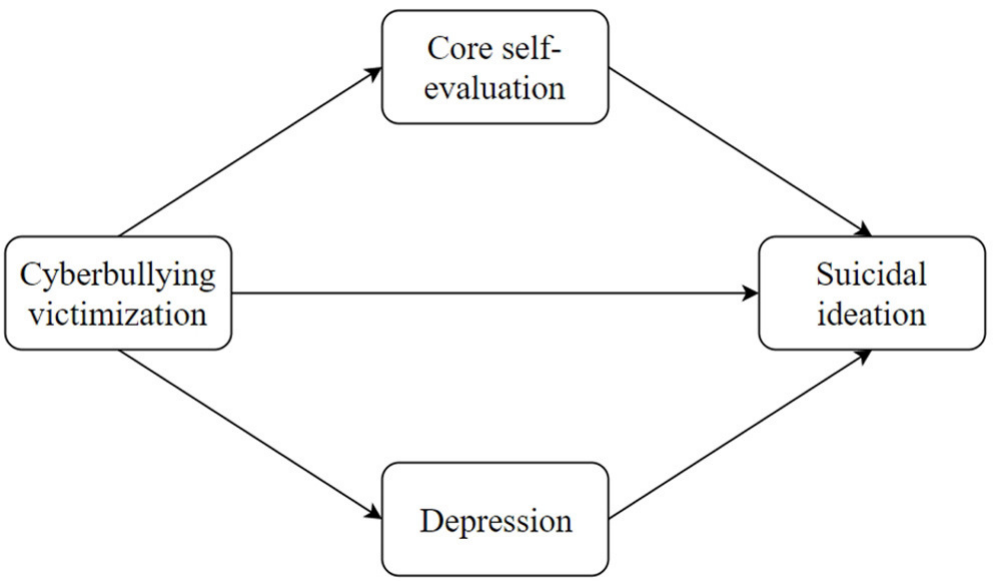
Proposed conceptual model.

In brief, the present study constructed a parallel mediation model to examine core self-evaluation and depression as two parallel mediators in the relationship between cyberbullying victimization and suicidal ideation (see Figure 1). This study hypothesized that core self-evaluation and depression would parallelly mediate the relationship between cyberbullying victimization and suicidal ideation.

## Methods

### Participants

A convenience sampling method was used to select participants from vocational colleges in Zhejiang and Anhui provinces for the survey. A total of 9,091 students participated in the project about career and psychological development of vocational university students in China. This study was a part of the project. Questionnaires with inattentive responses (e.g., those with <10 min of answer time and those with identical or regular answers) were removed, leaving 7,485 valid cases. For the purpose of avoiding overvaluing the association between variables on account of the large sample size, 20% of the total sample was randomly selected in this study according to the G^*^Power's guidelines (*n* = 1,289). Finally, 1,509 cases were obtained in the following analysis. Among them, 686 (45.5%) were male students, and 823 (54.5%) were female students. Their ages spanned from 16 to 25 years (*M* = 19.06, *SD* = 1.04). In terms of family status, 88.6% of the students lived in two-parent families, and 11.4% lived in single-parent or remarried families. The students were asked to rate their family's economic status on a 10-point scale. The results showed a mean score of 5.127 (*SD* = 1.51) on this scale, which meant that the family economic status of this sample was at an intermediate level.

### Procedure

The Institutional Review Board of the authors' university approved this study. Participants were recruited at two vocational colleges after informed consent was obtained from the students. An online survey was conducted in a classroom in the presence of a mental health teacher or a researcher. The survey was generated by the Questionnaire Star, which is a popular online survey website in China. Teachers or researchers send the link of the website to the students. Since all survey items were set as mandatory questions on the Questionnaire Star website, no data were omitted from this study. After students finish submitting the questions, there would be a page of “successful submission.” To ensure all students completed the survey in class, we required each student to submit the screenshots of this page in the class Wechat group after completing the questionnaire. The voluntary nature of participation in the survey and the confidentiality of its results were first emphasized to the participants before the questionnaire was distributed to the students. The online survey was opened on October 18, 2021, and closed on October 31, 2021. During these 2 weeks, no one except participants from the target universities visited the websites.

### Measures

#### Cyberbullying Victimization (CV)

Cyberbullying victimization was assessed using the victimization subscale of the Chinese version of the Revised Cyberbullying Inventory (45). The subscale consists of 14 activities about cyberbullying victimization (e.g., “People sent threatening or hurtful text messages to me”). Each participant rated how often he or she had engaged in those cyberbullying victimization behaviors during the previous 6 months on a 4-point scale (1 = *never*, 4 = *more than three times*). The mean score of each participant was calculated, and the higher values represented a higher incidence of cyberbullying victimization. The Cronbach's alpha coefficient for the cyberbullying victimization subscale in this study was 0.957.

#### Core Self-Evaluation (CSE)

Core self-evaluation was assessed using the Chinese version (46) of the Core Self-Evaluation Scale (47). The subscale consists of 10 items (e.g., “I am capable of coping with most of my problems”) (6 items are scored in reverse). Each participant rated the extent to which he or she agree or disagree on a 5-point scale (1 = *strongly disagree*, 5 = *strongly agree*). The mean score of each participant was calculated, and the higher values represented a higher level of core self-evaluation. The Cronbach's alpha coefficient for the Chinese version of the Core Self-Evaluation Scale in this study was 0.824.

#### Depression

Depression was assessed using the Chinese version (48) of the Center for Epidemiologic Studies Depression Scale (49). The subscale consists of 20 questions about depressive symptoms (e.g., “I had trouble keeping my mind on what I was doing”) (4 items are scored in reverse). Each participant rated the extent to which he or she has experienced the depressive symptoms during the last week on a 4-point scale (1 = *never*, 4 = *always*). The mean score of each participant was calculated, and the higher values represented a higher level of depression. The Cronbach's alpha coefficient for this scale in this study was 0.889.

#### Suicidal Ideation

Suicidal ideation was assessed using Beck Scale for Suicide Ideation-Chinese Version [BSI-CV; (50)]. The subscale consists of 5 questions about suicidal ideation (e.g., “To what extent do you wish to die”) (2 items are scored in reverse). Each participant rated their levels of agreement with the questions during the latest week on a 3-point scale (1 = *never*, 3 = *always*). The mean score of each participant was calculated, and the higher values represented a higher level of suicidal ideation. The Cronbach's alpha coefficient for this scale in the present study was 0.697.

## Data Analyses

All data analyses were performed using the SPSS 25.0 software package. Pearson's correlation analysis was used to explore potential relationships between cyberbullying victimization, core self-evaluation, depression, and suicidal ideation. The parallel mediation model was then tested using the SPSS macro PROCESS (51). This PROCESS has been widely used to test simple and complex mediating models (52, 53). In this study, model 4 was used to test the mediated models. The 95% bias-corrected confidence intervals for indirect effects were estimated from 5,000 resamples. When the confidence intervals (CIs) did not include zero, the indirect effects were significant.

## Results

### Descriptive and Correlational Analyses

The descriptive statistics between variables were presented in Table 1. Correlation analysis was performed on the mean scores of each variable (see Table 1). The research variables were significantly correlated in the predicted direction. Cyberbullying victimization was negatively correlated with core self-evaluation (*r* = −0.164, *p* < 0.001) and positively correlated with depression (*r* = 0.248, *p* < 0.001) and suicidal ideation (*r* = 0.287, *p* < 0.001). Core self-evaluation was negatively correlated with depression (*r* = −0.543, *p* < 0.001) and suicidal ideation (*r* = −0.340, *p* < 0.001). Depression was positively associated with suicidal ideation (*r* = 0.413, *p* < 0.001).

**Table 1:** Descriptive statistics and intercorrelations between variables.

**Variables**	**1**	**2**	**3**	**4**	**5**	**6**	**7**	**8**
1. Gender								
2. Age	0.026							
3. Family status	0.034	0.035						
4. SES	0.026	−0.046	−0.116^**^					
5. Cyberbullying victimization	−0.155^**^	0.014	0.005	−0.029				
6. CSE	−0.003	0.030	−0.061^*^	0.133^**^	−0.164^**^			
7. Depression	−0.047	0.030	0.054^*^	−0.098^**^	0.248^**^	−0.543^**^		
8. Suicidal ideation	−0.042	−0.026	0.025	−0.102^**^	0.287^**^	−0.340^**^	0.413^**^	
*M*	0.545	19.055	0.114	5.127	1.209	3.288	2.039	1.231
*SD*	0.498	1.045	0.318	1.509	0.464	0.492	0.375	0.341

*N = 1,509. Gender and family status were dummy coded (male = 0, female = 1; two-parent family = 0, single-parent and remarried family = 1). SES, Socioeconomic status; CSE, Core self-evaluation*.

* *p < 0.05*.

** *p < 0.001*.

### Testing for the Proposed Parallel Mediation Model

**Figure 2:**
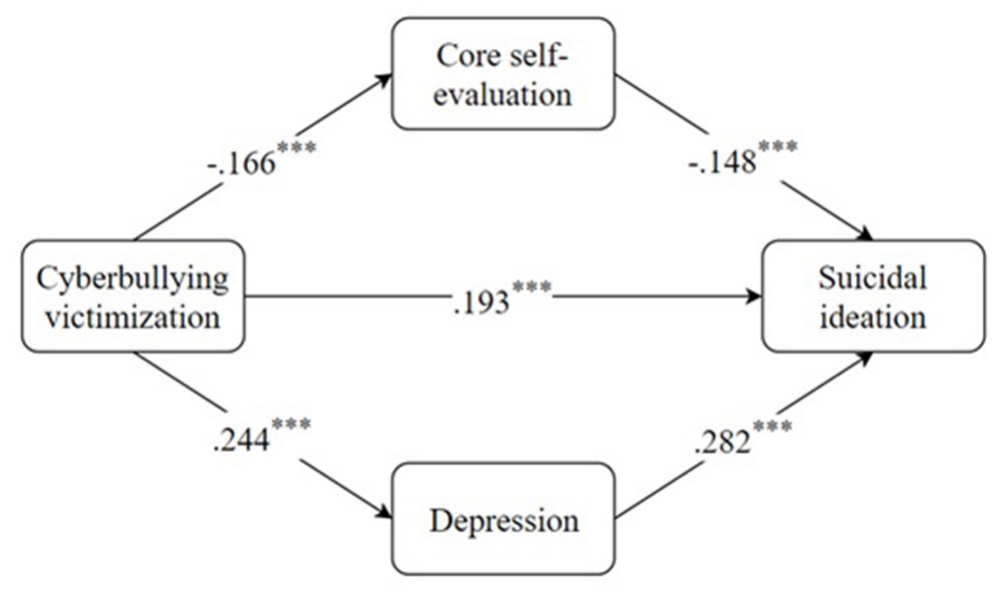
Standardized path coefficients for the pararlel mediation model. ***P < 0.001.

We utilized three regression models to examine the hypothesized parallel mediation model. The results were displayed in Table 2. In these equations of regression, participants' gender, age, family status, and social-economic status were controlled. As depicted in Figure 1, two indirect paths need to be checked: (1) CV → CSE → suicidal ideation; (2) CV → depression → suicidal ideation. About the first indirect route, CV was negatively associated with CSE (β = −0.166, *p* < 0.001), and CSE was negatively associated with suicidal ideation (β = −0.148, *p* < 0.001). The direct relationship between CV and suicidal ideation was also significant (β = 0.193, *p* < 0.001). This suggested that CSE played a partially mediating role between CV and suicidal ideation. About the second indirect route, CV was positively associated with depression (β = 0.244, *p* < 0.001), and depression was positively associated with suicidal ideation (β = 0.282, *p* < 0.001). The direct relationship between CV and suicidal ideation was also significant (β = 0.193, *p* < 0.001). This suggested that depression played a partially mediating role between CV and suicidal ideation. For clarity, the standardized coefficients for each path in the parallel mediation model were displayed in Figure 2.

**Table 2:** Regressions testing core self-evaluation and depression as parallel mediators in the relationship between cyberbullying victimization and suicidal ideation.

**Regression models**	**β**	***SE***	***t* value**	**LLCI**	**ULCI**	***R* ^2^**	***F* value**
**Model 1**						0.048	15.175^**^
Outcome: CSE							
Predictors: CV	−0.166	0.026	−6.513^**^	−0.216	−0.116		
Gender	−0.032	0.051	−1.240	−0.164	−0.037		
Age	0.041	0.025	1.625	−0.009	0.091		
Family status	−0.047	0.080	−1.832	−0.303	0.010		
SES	0.125	0.025	4.926^**^	0.075	0.175		
**Model 2**						0.072	23.327^**^
Outcome: Depression							
Predictors: CV	0.244	0.025	9.678^**^	0.194	0.293		
Gender	−0.009	0.051	−0.364	−0.118	0.081		
Age	0.021	0.025	0.842	−0.028	0.070		
Family status	0.043	0.079	1.707	−0.020	0.289		
SES	−0.085	0.025	−3.372^**^	−0.134	−0.035		
**Model 3**							
Outcome: Suicidal ideation						0.228	63.266^**^
Predictors: CV	0.193	0.024	8.121^**^	0.151	0.245		
CSE	−0.148	0.027	−5.427^**^	−0.340	−0.245		
Depression	0.282	0.028	10.218^**^	0.313	0.406		
Gender	0.003	0.046	0.129	−0.072	0.110		
Age	−0.034	0.023	−1.512	−0.078	0.011		
Family status	−0.005	0.072	−0.208	−0.150	0.132		
SES	−0.052	0.023	−2.234^*^	−0.098	−0.007		

*N = 1,509. Gender and family status were dummy coded (male = 0, female = 1; two-parent family = 0, single-parent and remarried family = 1). CV, Cyberbullying victimization; CSE, Core self-evaluation; SES, Socioeconomic status; LLCI, Lower limit of confidence interval; ULCI, Upper limit of confidence interval. The research variables (excluding gender and family status) in regression models were standardized*.

* *p < 0.05*.

** *p < 0.001*.

As shown in Table 3, the mediating effect of cyberbullying victimization on suicidal ideation *via* core self-evaluation was significant [Effect = 0.025, Boot SE = 0.005, 95% CI = (0.015, 0.035)]. The mediating effect of cyberbullying victimization on suicidal ideation *via* depression was significant [Effect = 0.069, Boot SE = 0.009, 95% CI = (0.052, 0.088)]. The results further showed that the mediation effect *via* core self-evaluation was weaker than the mediation effect *via* depression (β = −0.004, Boot SE = 0.011, 95% CI = (−0.067, −0.023)].

**Table 3:** Indirect effects with core self-evaluation and depression as mediators.

**Model**	**Effect**	**Boot SE**	**Boot 95% CI**		**Ratio**
			**Boot LLCI**	**Boot ULCI**	
Total indirect effect	0.093	0.010	0.075	0.113	33%
CV → CSE → suicidal ideation	0.025	0.005	0.015	0.035	9%
CV → depression → suicidal ideation	0.069	0.009	0.052	0.088	24%
CSE—depression	−0.044	0.011	−0.067	−0.023	–

*CV, Cyberbullying victimization; CSE, Core self-evaluation; LLCI, Lower limit of confidence interval; ULCI, Upper limit of confidence interval*.

## Discussion

Some studies have indicated that cyberbullying victimization is a risk factor for suicide. However, it remains unclear about the question of how cyberbullying victimization predicts suicidal ideation. To address this question, the present study constructs a parallel mediation model to analyze the relationship between cyberbullying victimization and suicidal ideation through core self-evaluation and depression. Results indicate that core self-evaluation and depression separately mediate the relationship between cyberbullying victimization and suicidal ideation. The mediating effect of depression is stronger than that of core self-evaluation. The parallel mediation model extends the knowledge of the mechanism underlying the relationship between cyberbullying victimization and suicidal ideation and provides suggestions for interventions on suicidal ideation among college students who have suffered from cyberbullying.

Consistent with previous research (7, 54–56), the present study also demonstrates cyberbullying victimization as a positive predictor of suicidal ideation. In general, individuals have a basic psychological need to fit in and be accepted by their peer group (57). When they suffer from cyberbullying, they feel abandoned by the group, which may lead to depressed moods and suicidal ideation. Moreover, college students are still in the ivory tower of school and have less social experience. When they experience stressful events such as cyberbullying, they are more likely to respond in a negative way (58), and feel fear and frustration, which in turn may trigger their internalizing problem behaviors, such as suicidal ideation.

The findings support the mediating role of core self-evaluation in the relationship between cyberbullying victimization and suicidal ideation. The hypothesis is supported. Our findings support the PMF (18), suggesting that negative events affect individuals' psychological adaption by influencing their cognition. That is, when individuals experience negative events such as cyberbullying, they are prone to non-adaptive cognitions that subsequently affect their psychological and behavioral adaptation (19). When students are in a cyberbullying situation, they feel hostility and discrimination from others and the world, so they are likely to experience frustration, reduce their most basic evaluation of their abilities and values, and then lower their core self-evaluation. At the same time, when college students' core self-evaluation is low, they tend to fall into negative emotions and believe that they are incapable of controlling the events that occur, so they are more inclined to adopt negative coping strategies, such as suicidal ideation (59). It has been suggested that individuals with low core self-evaluations experience more stress and tension in adverse situations and are more likely to use avoidance coping strategies (60). This study broadens the previous research by focusing more on the mediating effect of psychological factors (e.g., core self-evaluation) in the bullying victimization-suicide relationship. This reminds schools and families to pay attention to children's basic evaluations of themselves, so that the negative outcomes associated with cyberbullying victimization may be prevented.

Furthermore, the findings support the mediating role of depression in the relationship between cyberbullying victimization and suicidal ideation. This is consistent with the findings of Bauman et al. (61). As a repetitive, chronic, and uncontrollable stressor, cyberbullying victimization may evoke individuals' levels of depression (Chu et al., 2018) (32–34, 62). According to the behavioral theory of depression (63, 64), which holds that depression is a response to stressful events beyond the victim's control in life, when an event is beyond the victim's control (e.g., being bullied), the victim may feel helpless. After a long cycle, it is possible to lead to learned helplessness, which will eventually aggravate the depression. It is known that depressed individuals who are suffered from cyberbullying face many difficulties in interpersonal relationships, such as lacking a sense of belonging (65). According to the interpersonal theory of suicide (43), thwarted belonging may increase the risk of suicidal thoughts. In contrast to previous studies uncovering the relationship between cyberbullying victimization and suicidal ideation among secondary school students (7, 54–56), the present study proves this association among college students. Our findings broaden the age range of previous findings on the relationship between cyberbullying victimization and suicidal ideation among adolescents. Future work could investigate this possibility by utilizing longitudinal data.

Additionally, the mediating effect of depression is stronger than that of core self-evaluation. In China, with the rapid social and economic development, the competition in education and work is becoming more and more intense. College students are in the bridging time period between the end of their studies and their entry into the workplace, so college students are under more pressure to achieve a better life in the future. As a result, the pressure on Chinese college students is considerable. A meta-analysis has shown that the overall prevalence of depression among Chinese college students is 26.0%, and the number is still rising (66). In addition, influenced by Chinese culture, Chinese people pay more attention to the economic level as well as social status and relatively ignore individual mental health problems, such as depression. Therefore, when being bullied for long periods of time, individuals are more likely to develop depression, which has the potential to increase the frequency of suicidal ideation. The results of this study suggest that families, schools, and society should be concerned about individuals' mental health.

The results of this study may provide some intervention insights for students, teachers, parents, and researchers in responding to negative outcomes of cyberbullying victimization. The results suggest that low core self- evaluation and depression deserve more attention, which are all vulnerable factors for adolescents to develop suicidal ideation. It is recommended that families and schools could help victims to build self-acceptance and self-affirmation by using methods such as self-assertiveness training and cognitive reconstruction training. It is best to focus on intervention programs that can specifically work to improve core self-evaluation and reduce depression for victims of cyberbullying. The cognitive behavioral therapy (CBT) is one of the prevention programs that modifies maladaptive cognitions (e.g., low core self-evaluation) and thereby reduces emotional distress (e.g., depression) (67). It has been studied and proven to be effective in treating depression (68).

However, there are still some limitations. First, our sample was recruited in Zhejiang and Anhui provinces in China, thus limiting the generalizability of the results to other countries. Second, the present study uses a cross-sectional design, so the causal relationships could not be determined between cyberbullying victimization, core self-evaluation, depression, and suicidal ideation. Future research could pursue longitudinal design to examine the direction of the relationships among the research variables. Third, the use of self-reporting may not exclude inattentive responses, and there is also a social approval effect. Future studies should use alternative methods to examine conceptual models, such as using multiple informants (e.g., parents, teachers, and peers).

To conclude, this study extends our knowledge about the mechanisms linking cyberbullying victimization to suicidal ideation in college students. Core self-evaluation and depression are two mediators that may explain *why* these two constructs are associated with each other. The present findings indicate that core self-evaluation and depression separately mediate the relationship between cyberbullying victimization and suicidal ideation. In addition, this study also indicate that the mediating effect of depression is stronger than that of core self-evaluation. This suggests that we need to pay attention to individual mental health issues, such as depression. Our study may help to develop interventions and prevention measures for individuals' cyberbullying victimization in terms of core self-evaluation and depression.

## Data Availability Statement

The raw data supporting the conclusions of this article will be made available by the authors, without undue reservation.

## Ethics Statement

The studies involving human participants were reviewed and approved by IRB, Institute of Psychological and Brain Sciences, Zhejiang Normal University. Written informed consent to participate in this study was provided by the participants' legal guardian/next of kin.

## Author Contributions

XC: investigation, writing—original draft, and funding acquisition. SY, ZS, MJ, and RX: writing—review and editing. All authors contributed to the article and approved the submitted version.

## Funding

This work was supported by the National Social Science Foundation of China (Project No. CBA210234).

## Conflict of Interest

The authors declare that the research was conducted in the absence of any commercial or financial relationships that could be construed as a potential conflict of interest.

## Publisher's Note

All claims expressed in this article are solely those of the authors and do not necessarily represent those of their affiliated organizations, or those of the publisher, the editors and the reviewers. Any product that may be evaluated in this article, or claim that may be made by its manufacturer, is not guaranteed or endorsed by the publisher.
